# Supplementary material for: SEC-seq: association of molecular signatures with antibody secretion in thousands of single human plasma cells
Source: Nat Commun. 2023 Jun 15;14:3567. doi: 10.1038/s41467-023-39367-8 (PMC10272111; doi:10.1038/s41467-023-39367-8)
Supplement: Supplementary file 1 — Supporting Information [file 41467_2023_39367_MOESM1_ESM.pdf]

SUPPLEMENTARY INFORMATION

AUTHORS LIST

Rene Yu-Hong Cheng*[1,2], Joseph de Rutte*[3,4], Cade Ellis K Ito[1,5], Andee R. Ott[1], Lucie Bosler[3], Wei-Ying Kuo[3], Jesse Liang[3], Brian E Hall[6], David J Rawlings[1,7,8], Dino Di Carlo**[3,4,9,10], Richard G. James**[1,2,5,8,11,12]

AFFILIATIONS

[1]Center of immunotherapy and Immunity, Seattle Children Research Institute, Seattle, WA 98101, USA,

[2]Molecular Engineering and Science Institute, University of Washington, Seattle, WA 98195, USA,

[3]Partillion Bioscience, Los Angeles, CA 90095, USA,

[4]Department of Bioengineering, University of California - Los Angeles, Los Angeles, CA 90095, USA,

[5]Department of Lab Medicine and Pathology, University of Washington, Seattle, WA 98195, USA,

[6]Luminex corporation, Seattle, WA 98119,

[7]Department of Immunology, University of Washington, Seattle, WA 98195, USA,

[8]Departments of Pediatrics, University of Washington, Seattle, WA 98195, USA,

[9]Department of Mechanical and Aerospace Engineering, Los Angeles, CA 90095, USA,

[10]California NanoSystems Institute (CNSI), University of California - Los Angeles, Los Angeles, CA 90095, USA,

[11]Department of pharmacology, University of Washington, Seattle, WA 98195, USA,
[12]Brotman-Baty Institute for Precision Medicine, Seattle, WA, 98195, USA.

*Contribute equally to this study.

**Corresponding authors: rickerj@u.washington.edu, dicarlo@ucla.edu

Supplementary Table 1. Buffer and reagents
Supplementary Table 2. Antibodies
Supplementary Table 3. ImageStreamX® MKII fluorochrome layout
Supplementary Table 4. ImageStreamX® MKII Collection Information
Supplementary Figures 1-13

Supplementary Table 1. Buffer and reagents

Buffer/Medium	Item	Vendor	Catalog	Concentration
Washing buffer	DPBS	Fisher Scientific	14190250	
	BSA	VWR life science	0332-100G	0.50%
	Pluronic F-127	Sigma	P2443-250G	0.05%
Staining buffer	DPBS	Fisher Scientific	14190250	
	FBS	Sigma	F0926-500ML	2%
	Pluronic F-127	Sigma	P2443-250G	0.05%
Sorting Buffer	PBS	Cytiva	SH30256.01	
	Blocker BAS (10%) in PBS	Thermo Scientific	37525	1%
	Pluronic F-127	Sigma	P2443-250G	0.05%
B cell loading medium	DMEM+GlutaMAX	Gibco	10569-010	
B cell secretion medium	Lonza BioWhittaker Iscove's Modified Dulbecco's Medium (IMDM) without L-Glutamine	Fisher	BW12726F	
	FBS	Sigma	F0926-500ML	10%
	GlutaMAX 100X	Gibco	35050-061	1X
	2-Mercaptoethanol	Fisher	O3446I-100	55um

Supplementary Table 2. Antibodies

Experiment	Item	Vendor	Catalog	Clone
human B cell capture antibody	Biotin anti-human CD45 Antibody	Biolegend	304004	HI30
human B cell capture antibody	Biotin anti-human CD38 Antibody	Biolegend	303518	HIT2
human B cell capture antibody	Biotin anti-human CD27 Antibody	Biolegend	356426	M-T271
human B cell capture antibody	Biotin anti-human CD31 Antibody	Biolegend	536604	O92E4
human IgG capture antibody/blocking antibody	Goat Anti-Human IgG Fc, Multi-Species SP ads-BIOT	Southen Biotech	2014-08	Polyclonal
Flow Cytometry	PE-Cy7 anti-human CD19	Biolegend	302216	HIB19
Flow Cytometry	AF700 anti-human CD138	Biolegend	356512	MI15
Flow Cytometry/Amnis	PerCP-Cy5.5 anti-human CD38	BD	BDB551400	HIT2
Flow Cytometry/Amnis	PE anti-human CD138	Biolegend	356504	MI15
Flow Cytometry/Amnis	PacBlue anti-human IgM	Biolegend	314514	MHM-88
Flow Cytometry	APC anti-human IgM	BD	551062	G20-127
Flow Cytometry	APC-Vio® 770 IgA Antibody, anti-human	Miltenyi	130-113-473	IS11-8E10
Flow Cytometry	PE anti-human IgG	BD	555787	G18-144
Flow Cytometry	AF700 anti-human IgG	BD	561296	G18-145

Flow Cytometry	APC anti-human CD59	Biolegend	304712	p282
Flow Cytometry	FITC anti-human CD98	Biolegend	315603	MEM-108
Amnis	APC anti-human IgG	BD	562025	G18-145
SEC-seq cross-sp	TotalSeq™-C0971 Streptavidin	Biolegend	405271	
SEC-seq cross-sp	anti-mouse CD45	R&D Systems	AF114	Polyclonal
SEC-seq cross-sp	Goat anti-Mouse IgG FC	Jackson Immuno Research	115-065-071	Polyclonal
SEC-seq cross-sp	TotalSeq™-C1167 anti-mouse IgG1 Antibody	Biolegend	406636	RMG1-1
SEC-seq	PE Mouse Anti-Human IgG	BD	555787	G18-145
SEC-seq	TotalSeq™-B0911 anti-phycoerythrin (PE) Antibody	Biolegend	408113	PE001

Supplementary Table 3. ImageStreamX® MKII fluorochrome layout

Ch	Band	Fluorochrome	Target	Notes
1	430-470	Brightfield	Morphology	FIXED
2	470-560			
3	560-595			
4	595-660	PE	CD138	Plasma cell marker
5	660-745	PerCP Cy5.5	CD38	Plasmablast/plasma cell marker
6	745-785	SSC	Granularity	
7	430-505	Pac Blue	IgM	Surface bound, not captured on nanovial
8	505-575			
9	575-595	Brightfield	Morphology	FIXED
10	595-660			
11	660-720	APC	IgG	Antibody released to nanovial by plasma B cells
12	720-785			

Supplementary Table 4. ImageStreamX® MKII Collection Information

Instrument: ISX 493	405 power: 120mw	488 power: 400mW	560 power: Off	
592 power: Off	642 power: 150mW	785 power: 2 mW	Mag: 20x	
INSPIRE: Current	Bin: None	Core diameter: 10	Velocity: 66	%Bd: 7

CameraSync: 38.9	Focus: 0.89	Core Tracking: 11	Focus Tracking: 0		

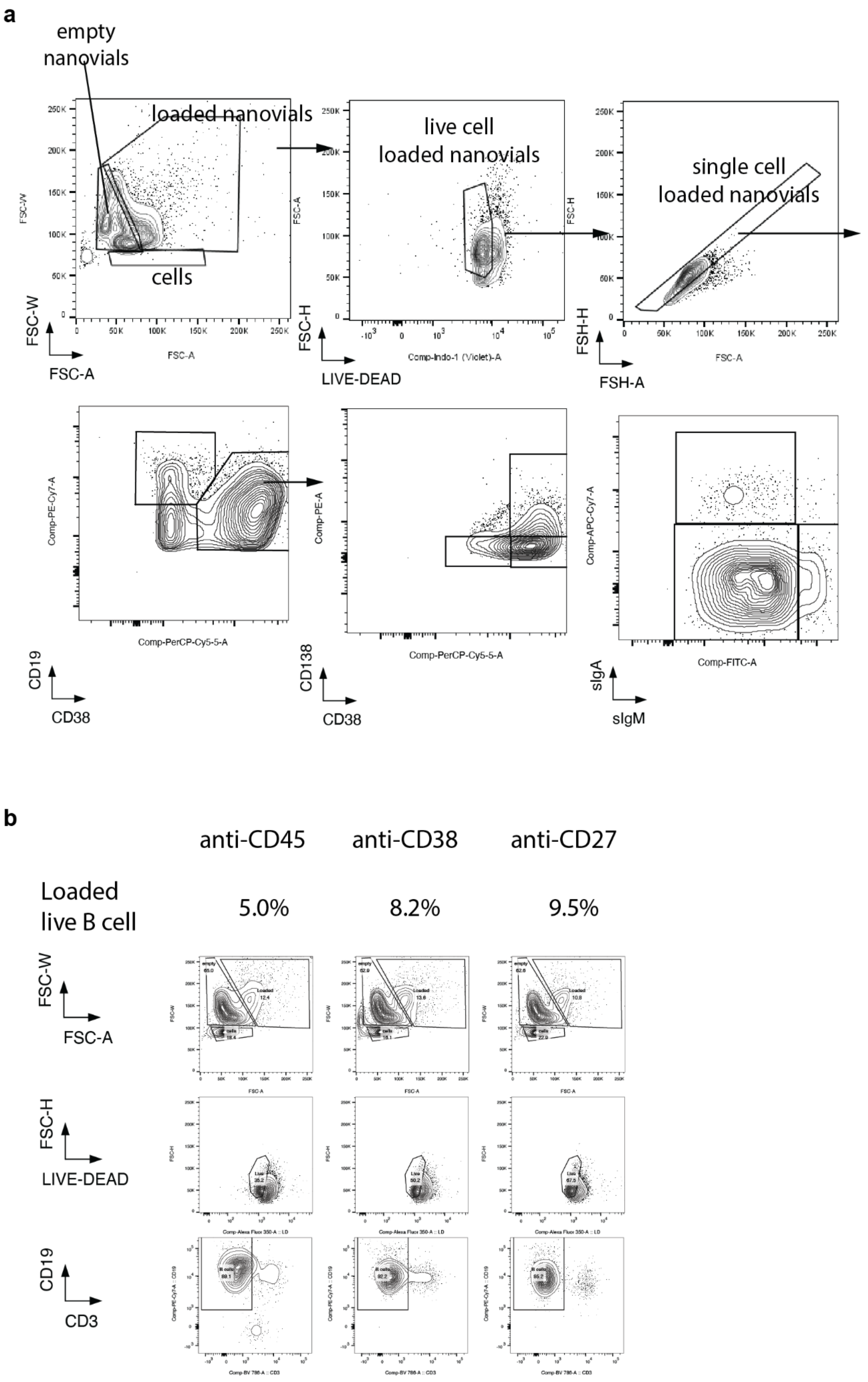
a
empty nanovials
loaded nanovials
cells
FSC-W
FSC-A
live cell loaded nanovials
FSC-H
LIVE-DEAD
Comp-Indo-1 (Violet)-A
single cell loaded nanovials
FSH-H
FSH-A
Comp-PE-Cy7-A
Comp-PerCP-Cy5-5-A
CD19
CD38
Comp-PE-A
CD138
Comp-APC-Cy7-A
Comp-FITC-A
sIgA
sIgM
b
anti-CD45
anti-CD38
anti-CD27
Loaded live B cell
5.0%
8.2%
9.5%
CD19
CD3

Supplementary Figure 1. Nanovial secretion assay flow cytometry gating strategy and characterization of cell loading using antibodies against different cell surface markers. **(a)** Gating strategy for live B cell loaded nanovials used for surface marker and IgG secretion analysis. **(b)** Flow plots of cell loading with different cell capture antibodies linked to the nanovials. Loaded live B cell percentage is calculated by dividing the loaded live B cell counts by the total number of nanovial counts (loaded nanovial counts + empty nanovial counts).

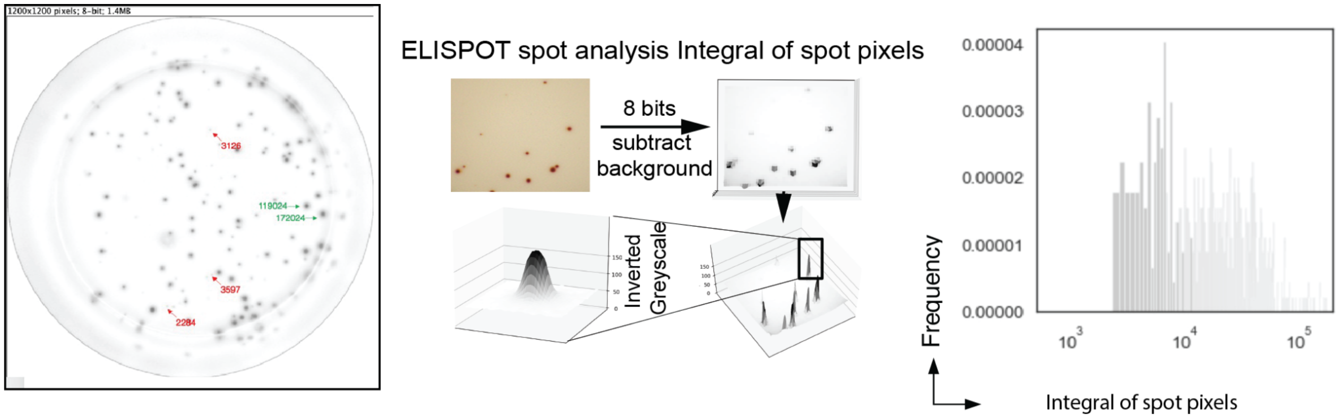

Supplementary Figure 2. ELISPOT spot analysis and IgG secretion heterogeneity.
An ELISPOT image (left) showing IgG secretion for plasma cells following our differentiation protocol. Red arrows point to events with low IgG secretion (small faint spots) and green arrows point to events with higher IgG secretion (larger, more intense spots). The spot intensity is integrated over the entire spot area using ImageJ and the output histogram (right) represents the heterogeneous IgG secretion ability of the population.

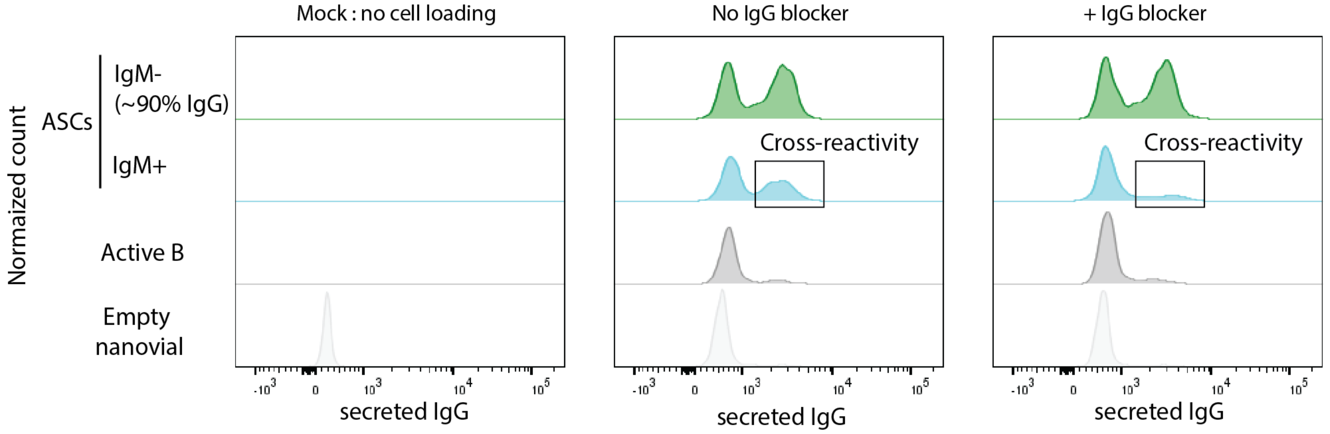

Supplementary Figure 3. Nanovial secretion with or without anti-IgG blocking.
We adjusted the loading strategy to reduce cross-reactive IgG signal to nanovials containing IgM cells. In order to reduce cross-reactive cells are incubated with anti-IgG as a sink for secreted IgG during the cell loading step and then the anti-IgG solution is washed prior to the cell secretion incubation step.

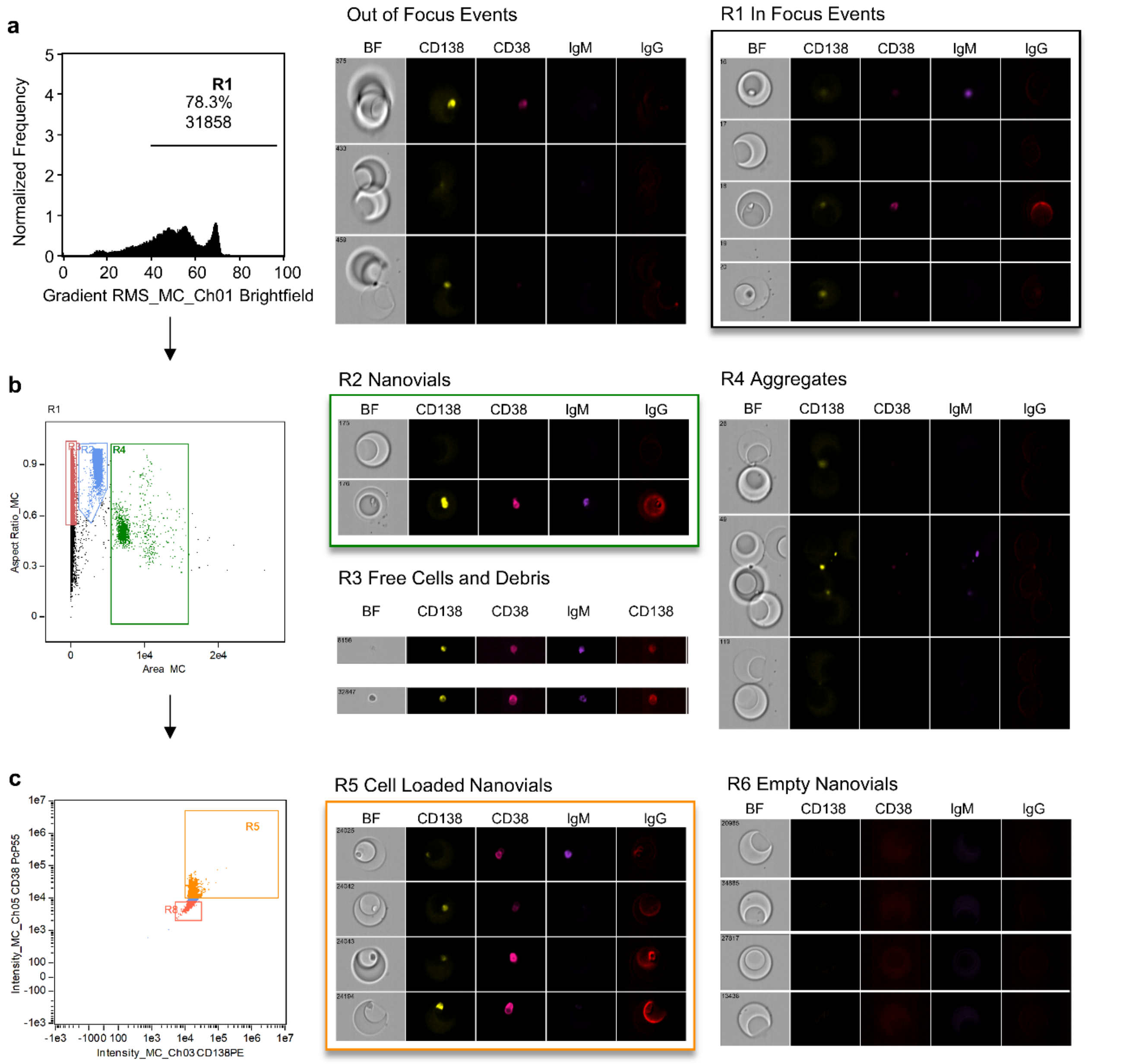

Supplementary Figure 4. Nanovial secretion assay gating strategy on the ImageStreamX® MKII. (a) Infocus events are identified by gating on the root mean square gradient of the brightfield channel (Gate R1). **(b)** Events associated with small debris, free cells, nanovials, and nanovial aggregates can be assigned based on the aspect ratio and area of the event. Gate R2 contains single nanovials. **(c)** Cell-loaded nanovials are then gated based on a threshold level of CD38 and CD138 signals (Gate R5).

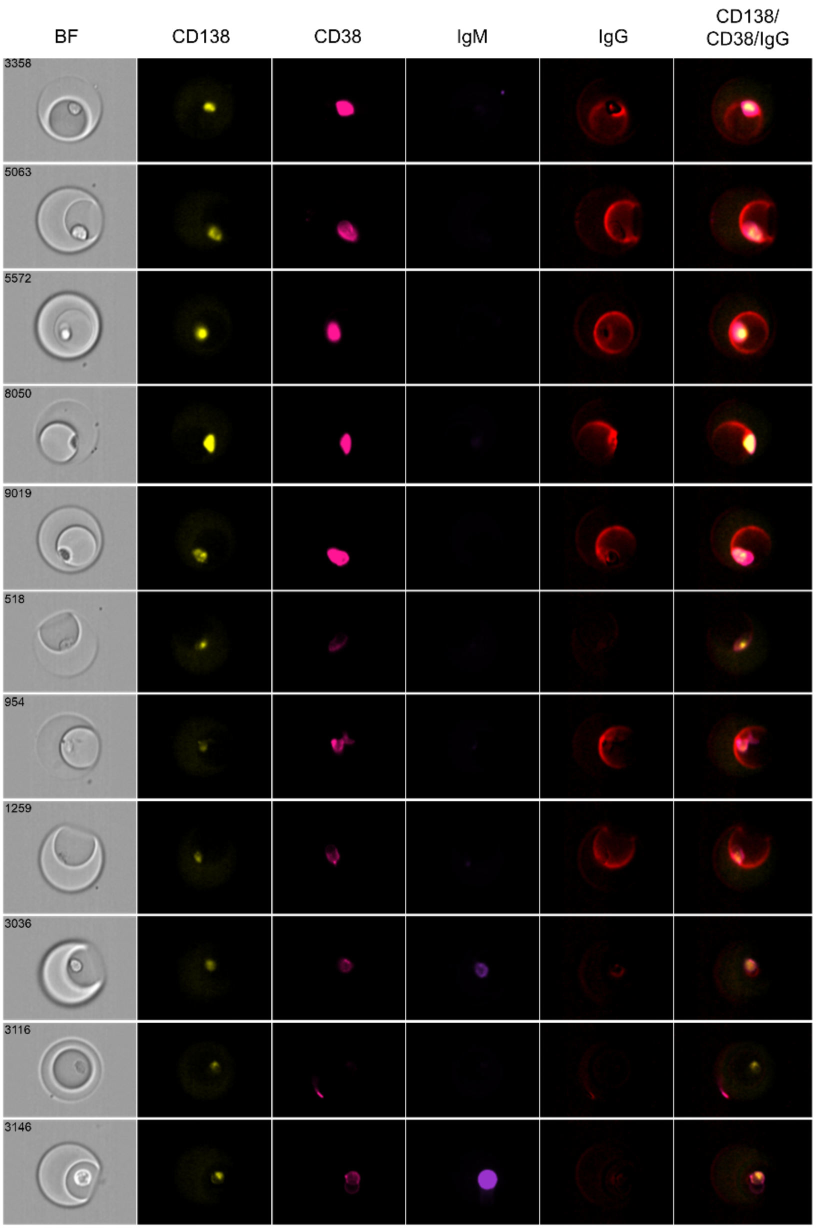

Supplementary Figure 5. Additional images of cell loaded nanovials taken with the ImageStreamX® MKII. Compared to the punctate cell surface signal for surface markers, the secreted IgG signal is clearly seen across the hemispherical cavity of the nanovial.

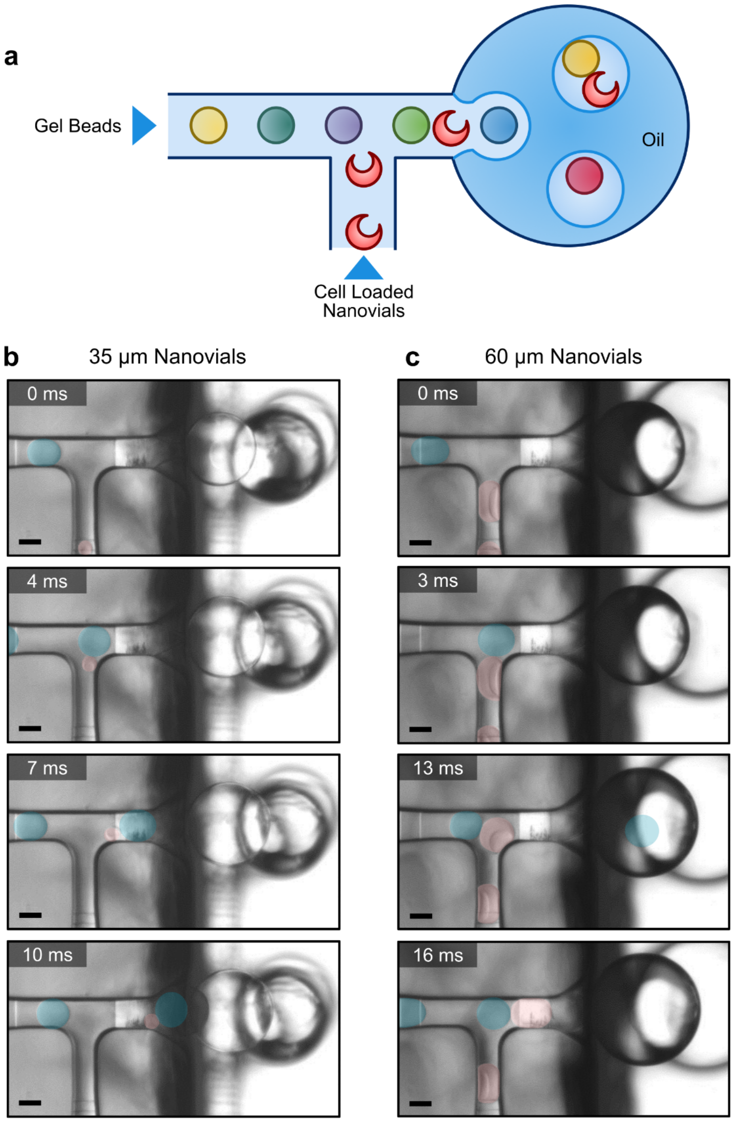

Supplementary Figure 6. High-speed microscopy images of the 10X Genomics gel beads and nanovials loading into droplets formed on the 10X Next GEM chip G. (a) Gel beads and nanovials are loaded through the gel bead inlet and cell sample inlets, respectively, as shown in the illustration. **(b)** 35 µm outer diameter nanovials pass through the cell sample inlet without obstruction and load into forming droplets. **(c)** 60 µm nanovials become constrained in the sample inlet and eventually pass through after deforming. Gel Beads and nanovials are false-colored blue and pink, respectively, to aid in visualization. Scale bar: 50 µm.

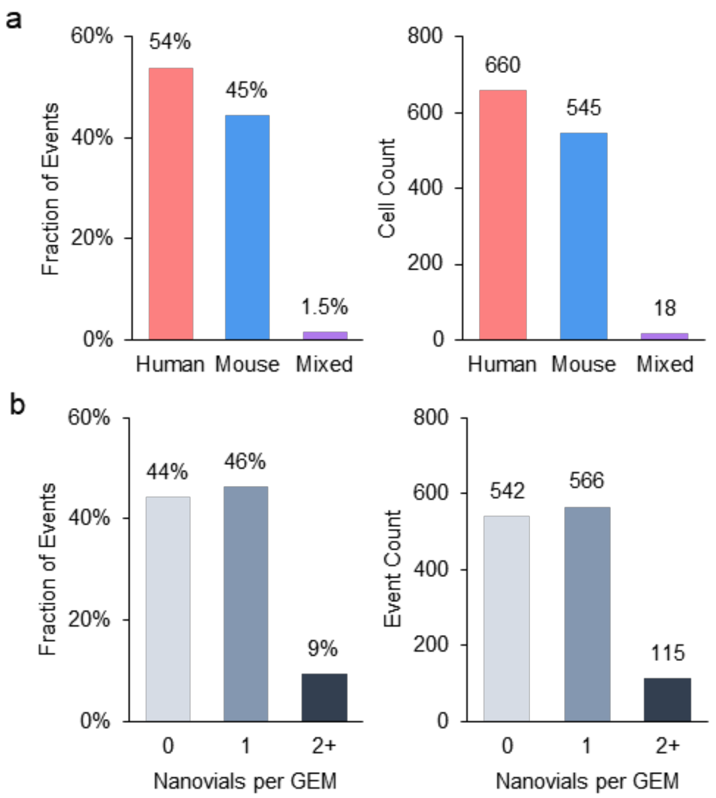

Supplementary Figure 7. Statistics on multiplet events from the mixed species experiments shown in figure 3. Nanovials were loaded into the 10X Chromium chip at the recommended concentration for cells to achieve 10,000 nanovial events. Approximately 10% of nanovials were loaded with cells and a roughly equal population of cells were off the nanovials. **(a)** The fraction of events based on species classification are plotted. The cell multiplet rate is close to the expected value reported in the 10X literature based on cell count (0.8% expected, 1.5% actual). **(b)** Fraction of events associated with 0, 1, or 2 or more nanovials. Using feature barcode reads on nanovials we discriminated the number of nanovials per cell event. Given there are ~10 times the number of nanovials as cells for this experiment the number of events with multiple nanovials are within the expected range (7.6% expected, 9% actual).

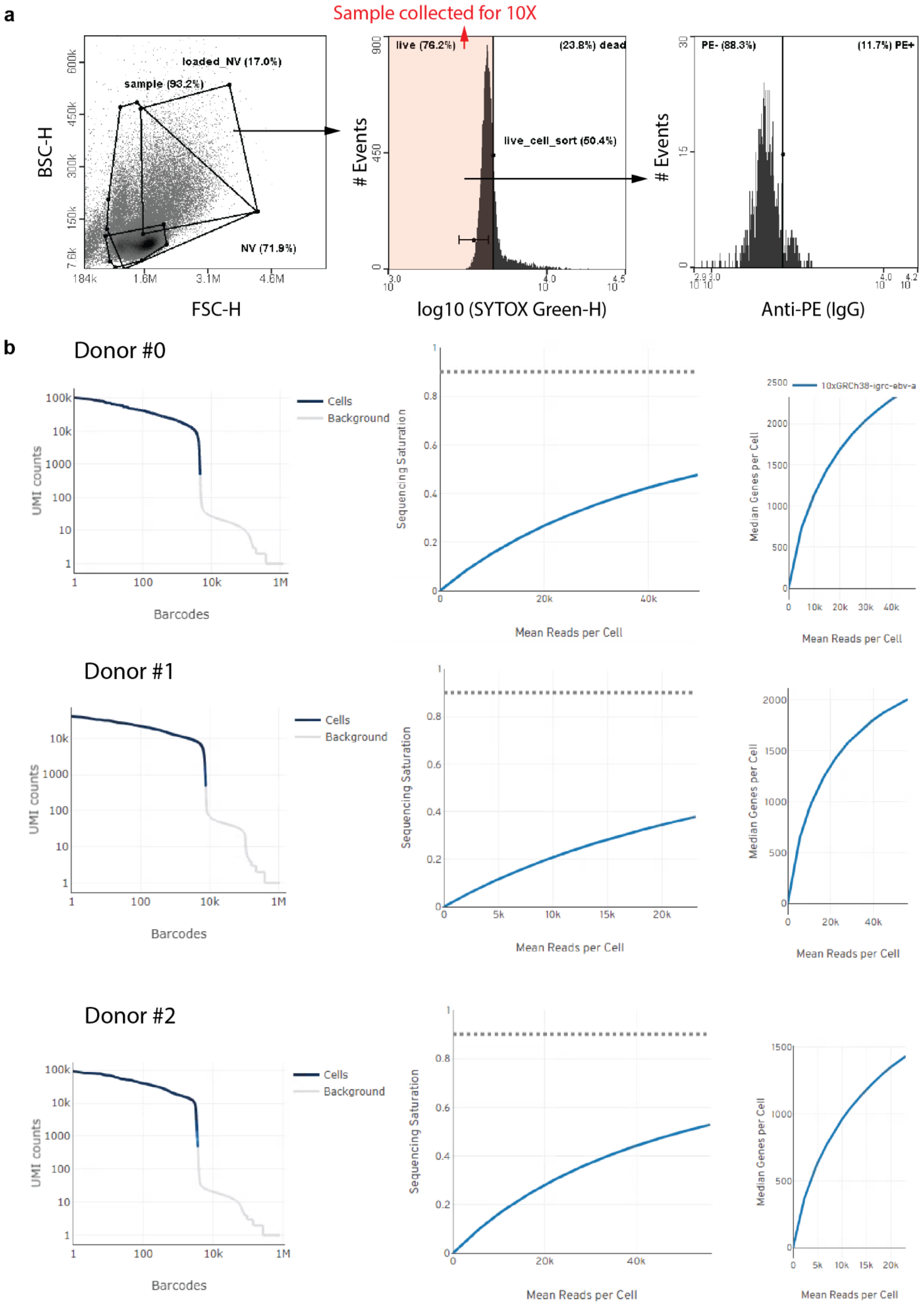

a
Sample collected for 10X
loaded_NV (17.0%)
sample (93.2%)
NV (71.9%)
BSC-H
FSC-H
live (76.2%)
(23.8%) dead
live_cell_sort (50.4%)
Events
log10 (SYTOX Green-H)
PE- (88.3%)
(11.7%) PE+
Anti-PE (IgG)
b
Donor #0
Donor #1
Donor #2
Cells
Background
UMI counts
Barcodes
Sequencing Saturation
Mean Reads per Cell
Median Genes per Cell
10xGRCh38-igrc-ebv-a

Supplementary Figure 8. Sorting live-cell-containing nanovials for SEC-seq and Cell Ranger QC

(a) Sorting strategy for live-cell-containing nanovials using the Nanocellect WOLF prior to loading into the 10X Chromium Next GEM chip. All live cells that were loaded on nanovials were collected independent of secretion amount. **(b)** 10X Cell Ranger QC summary from three donors (experiments). Barcode rank plot represents the UMI count separation between cells and background (left). Sequencing saturation plot shows the sequencing depth is ~50% by using a NextSeq 1000/2000 kit (middle).

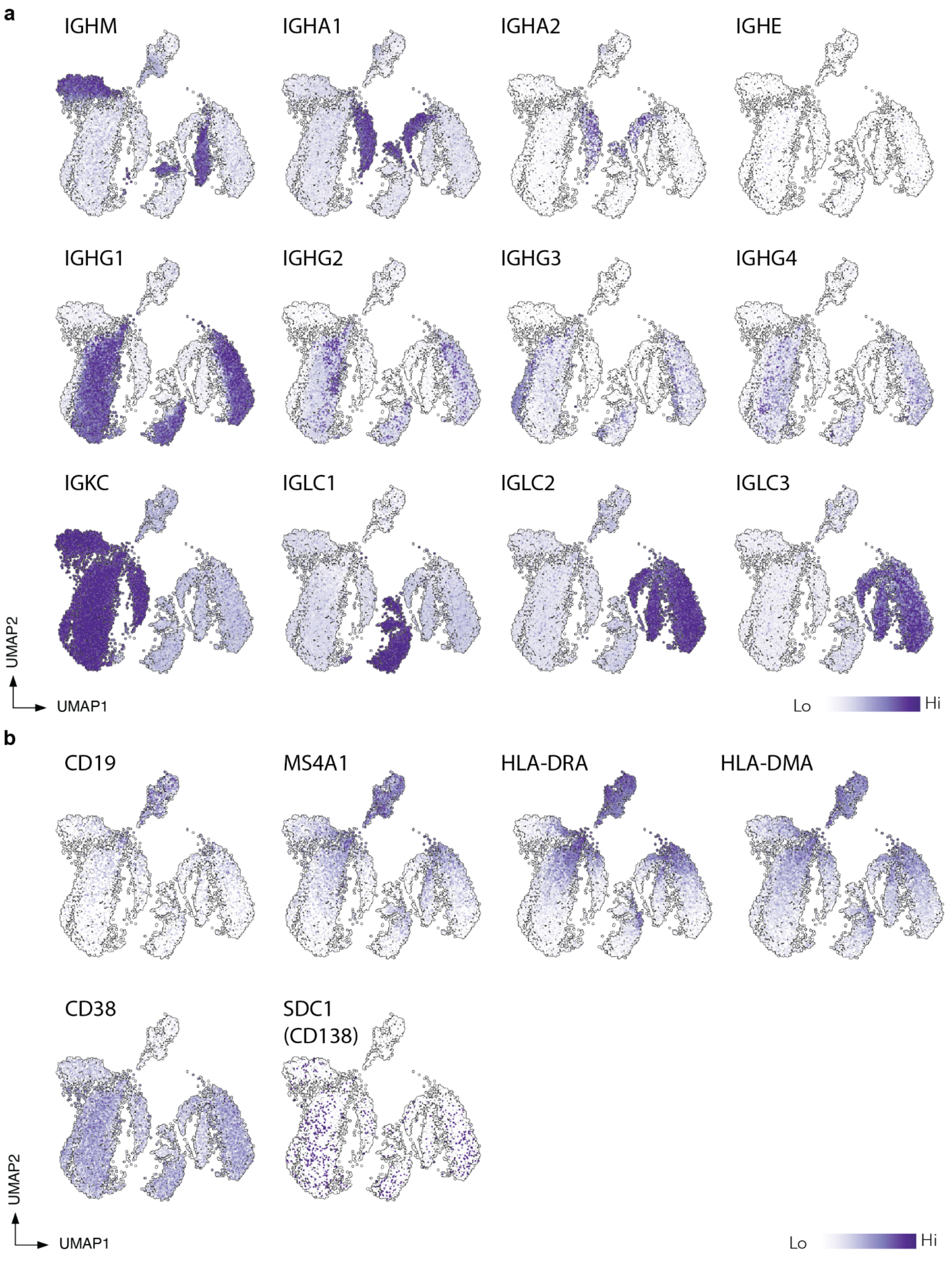
a
IGHM
IGHA1
IGHA2
IGHE
IGHG1
IGHG2
IGHG3
IGHG4
IGKC
IGLC1
IGLC2
IGLC3
UMAP2
UMAP1
Lo
Hi
b
CD19
MS4A1
HLA-DRA
HLA-DMA
CD38
SDC1
(CD138)
UMAP2
UMAP1
Lo
Hi

Supplementary Figure 9. UMAP of immunoglobulins and activated B cells/PC makers. Relative transcript levels of immunoglobulin genes projected on the UMAP plot from Fig 4b. **(a)** The top row represents non-IgG immunoglobulin classes, the middle row represents IgG subclasses, and the bottom row represents immunoglobulin light chain. **(b)** The top row represents activated B cell markers and the bottom row represents PC markers. All UMAP color bar are according to normalize expression data into unit of log transcripts.

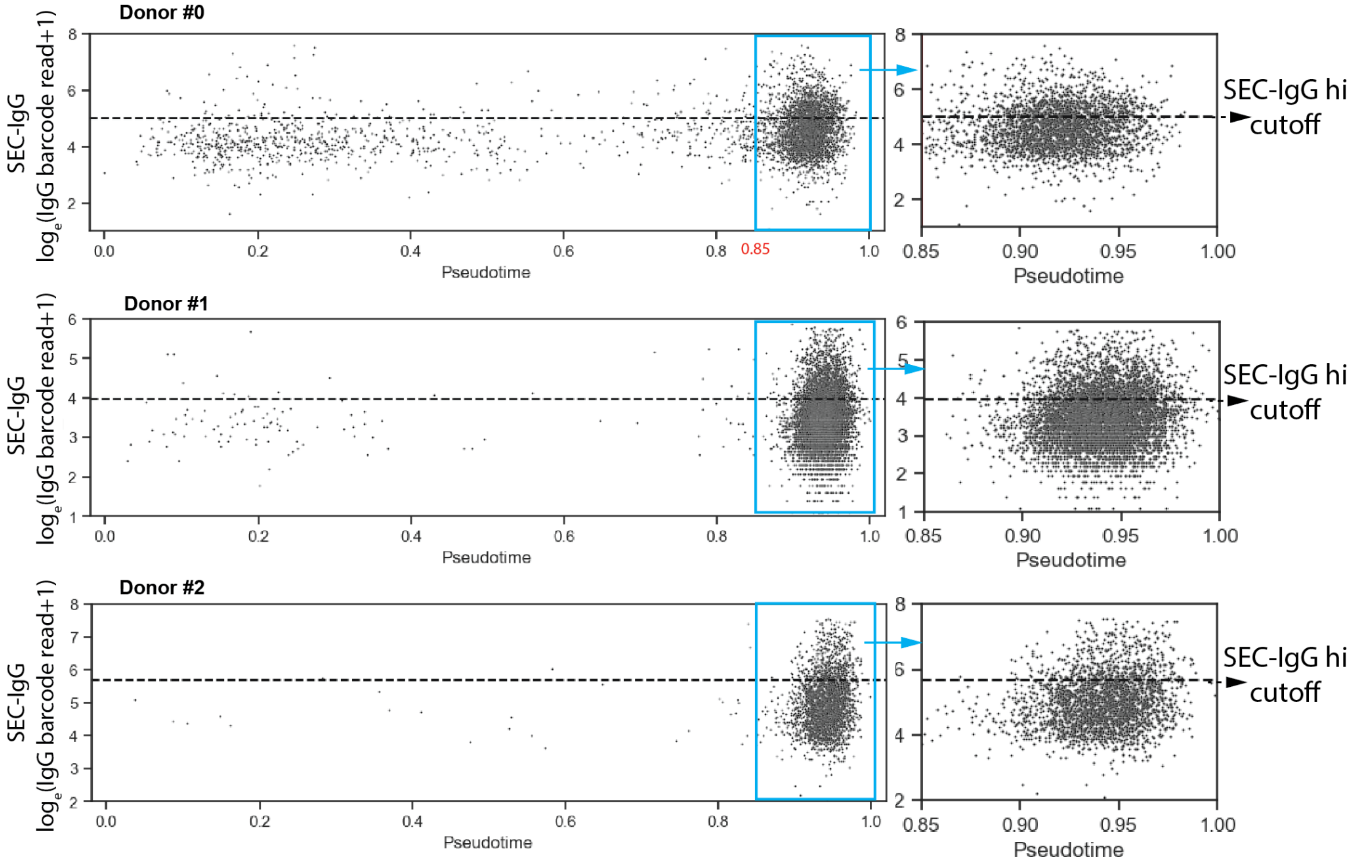

Supplementary Figure 10. Scatter plot showing the SEC-IgG barcode reads for each cell as a function of pseudotime. Data is plotted for each donor. The region above ~0.85 pseudotime contains the majority of cells and is expanded. Dash horizontal lines represent SEC-IgG hi cutoff from each donor.

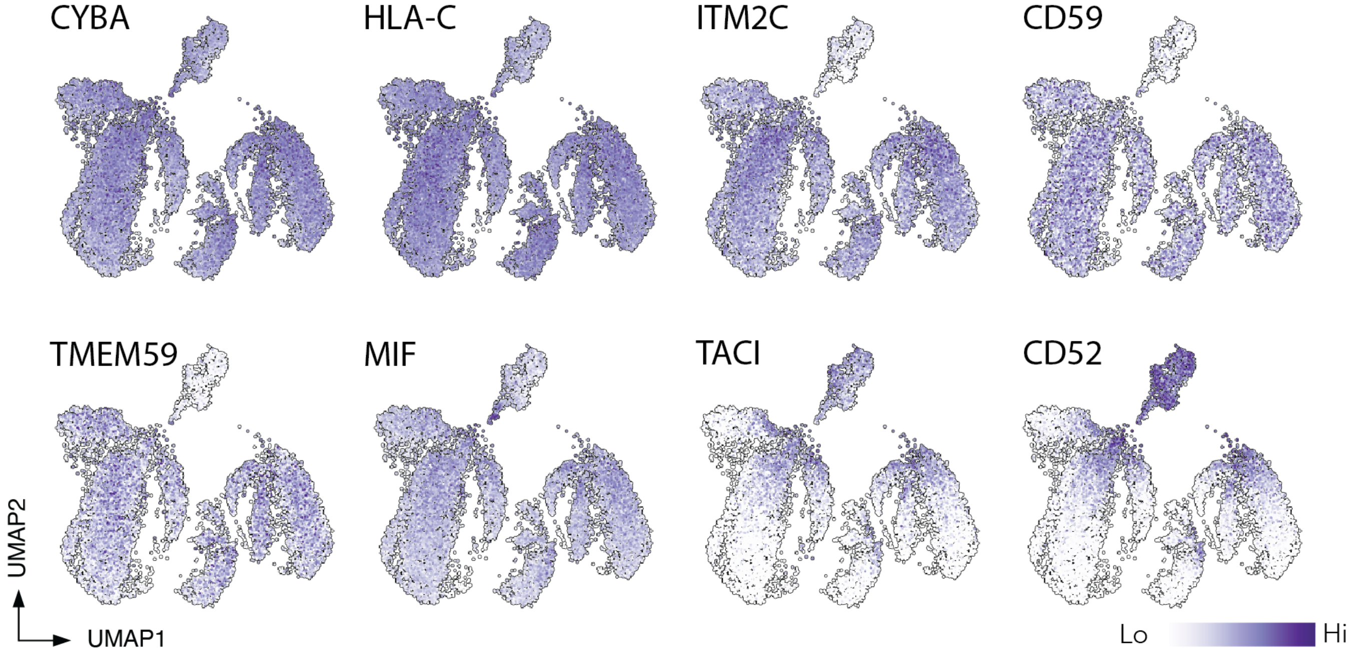

Supplementary Figure S11. UMAP of surface expressed genes correlated with SEC-IgG. Relative transcript levels of surface expressed genes projected on the UMAP plot from Fig 4b. All UMAP color bar are according to normalize expression data into unit of log transcripts.

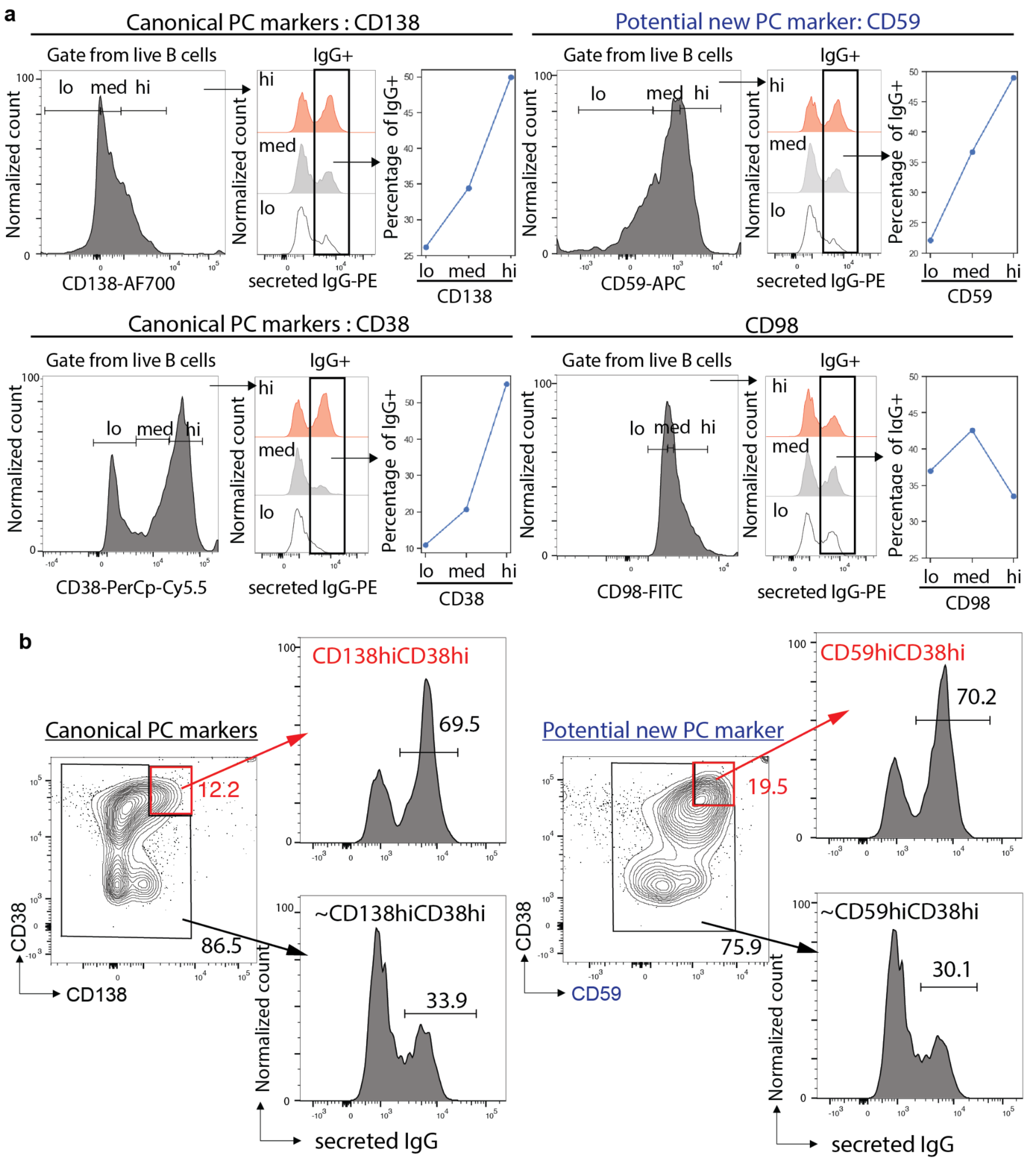

Supplementary Figure 12. Flow cytometry validation for surrogate PC marker, CD59. (a) Gating live B cells with low (lo), medium(med) and high(hi) expression of CD138, CD38, CD59 and CD98, and plot of percentage of IgG secretors from each gate. (b) Canonical gate for PC,

CD138hiCD38hi and histogram of secreted IgG from each indicated gate (left panel). Comparison with potential PC marker, CD59 (right panel).

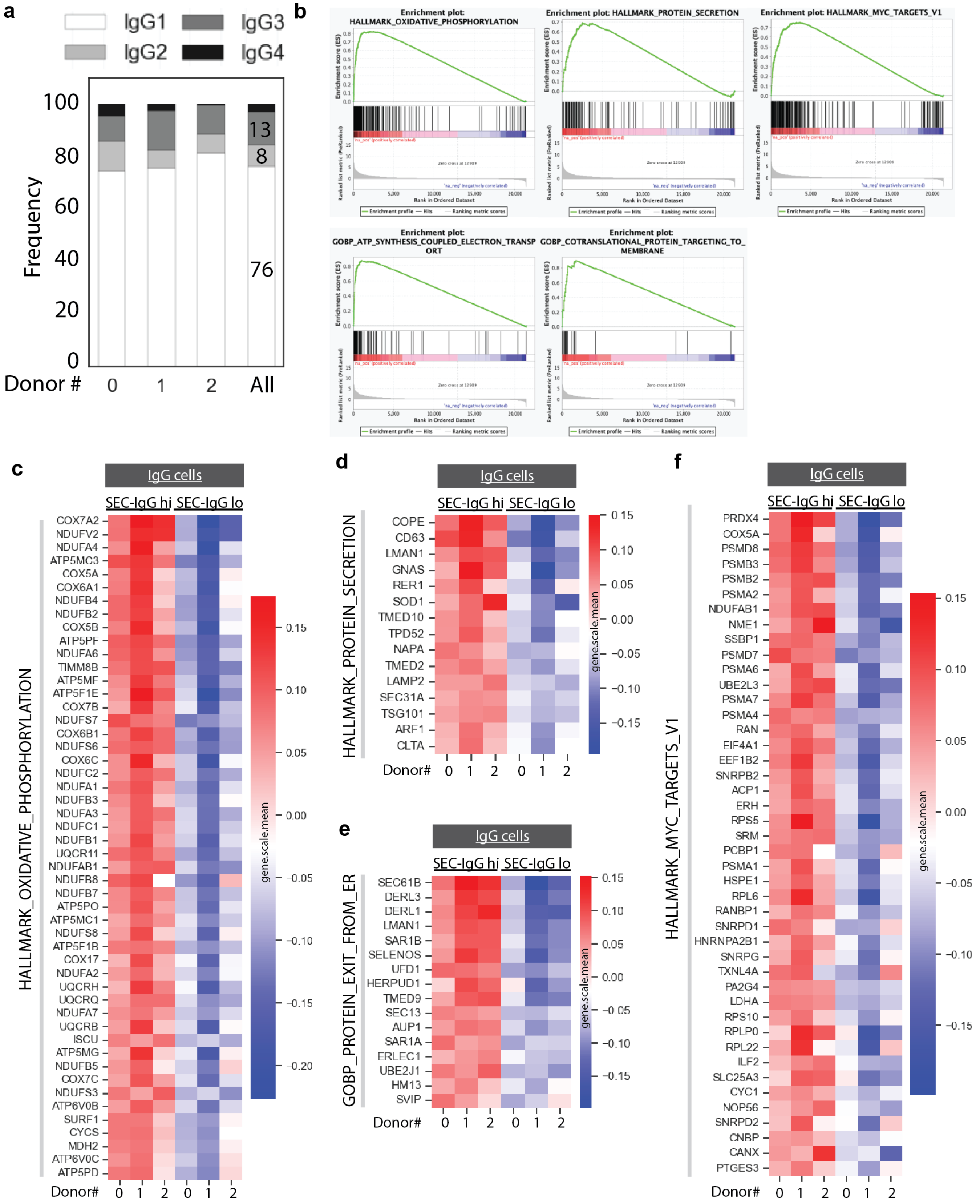
a
IgG1
IgG2
IgG3
IgG4
Frequency
100
80
60
40
20
0
13
8
76
Donor #
0
1
2
All
b
Enrichment plot: HALLMARK_OXIDATIVE_PHOSPHORYLATION
Enrichment plot: HALLMARK_PROTEIN_SECRETION
Enrichment plot: HALLMARK_MYC_TARGETS_V1
Enrichment plot: GOBP_ATP_SYNTHESIS_COUPLED_ELECTRON_TRANSPORT
Enrichment plot: GOBP_COTRANSLATIONAL_PROTEIN_TARGETING_TO_MEMBRANE
Enrichment score (ES)
Ranked list metric (PreRanked)
Rank in Ordered Dataset
Enrichment profile
Hits
Ranking metric scores
c
IgG cells
SEC-IgG hi
SEC-IgG lo
HALLMARK_OXIDATIVE_PHOSPHORYLATION
COX7A2
NDUFV2
NDUFA4
ATP5MC3
COX5A
COX6A1
NDUFB4
NDUFB2
COX5B
ATP5PF
NDUFA6
TIMM8B
ATP5MF
ATP5F1E
COX7B
NDUFS7
COX6B1
NDUFS6
COX6C
NDUFC2
NDUFA1
NDUFB3
NDUFA3
NDUFC1
NDUFB1
UQCR11
NDUFAB1
NDUFB8
NDUFB7
ATP5PO
ATP5MC1
NDUFS8
ATP5F1B
COX17
NDUFA2
UQCRH
UQCRQ
NDUFA7
UQCRB
ISCU
ATP5MG
NDUFB5
COX7C
NDUFS3
ATP6V0B
SURF1
CYCS
MDH2
ATP6V0C
ATP5PD
Donor#
0
1
2
0
1
2
gene.scale.mean
0.15
0.10
0.05
0.00
−0.05
−0.10
−0.15
−0.20
d
IgG cells
SEC-IgG hi
SEC-IgG lo
HALLMARK_PROTEIN_SECRETION
COPE
CD63
LMAN1
GNAS
RER1
SOD1
TMED10
TPD52
NAPA
TMED2
LAMP2
SEC31A
TSG101
ARF1
CLTA
Donor#
0
1
2
0
1
2
gene.scale.mean
0.15
0.10
0.05
0.00
−0.05
−0.10
−0.15
e
IgG cells
SEC-IgG hi
SEC-IgG lo
GOBP_PROTEIN_EXIT_FROM_ER
SEC61B
DERL3
DERL1
LMAN1
SAR1B
SELENOS
UFD1
HERPUD1
TMED9
SEC13
AUP1
SAR1A
ERLEC1
UBE2J1
HM13
SVIP
Donor#
0
1
2
0
1
2
gene.scale.mean
0.15
0.10
0.05
0.00
−0.05
−0.10
−0.15
f
IgG cells
SEC-IgG hi
SEC-IgG lo
HALLMARK_MYC_TARGETS_V1
PRDX4
COX5A
PSMD8
PSMB3
PSMB2
PSMA2
NDUFAB1
NME1
SSBP1
PSMD7
PSMA6
UBE2L3
PSMA7
PSMA4
RAN
EIF4A1
EEF1B2
SNRPB2
ACP1
ERH
RPS5
SRM
PCBP1
PSMA1
HSPE1
RPL6
RANBP1
SNRPD1
HNRNPA2B1
SNRPG
TXNL4A
PA2G4
LDHA
RPS10
RPLP0
RPL22
ILF2
SLC25A3
CYC1
NOP56
SNRPD2
CNBP
CANX
PTGES3
Donor#
0
1
2
0
1
2
gene.scale.mean
0.15
0.10
0.05
0.00
−0.05
−0.10
−0.15

Supplementary Figure 13. IgG isotypes and GSEA plot and gene list in core enrichment. **(a)** Bar graph representing the percentage of each isotype of IgG population from each donor (IgG1, IgG2, IgG3, and IgG4). **(b)** Representative GSEA plots of mitochondrial, protein transport and MYC-targets. **(c-f)** Top gene lists in core enrichment in **(c)** Hallmark oxidative phosphorylation, **(d)** Hallmark protein secretion **(e)** Biological process protein exit from ER, and **(f)** Hallmark MYC-targets. Heatmap represents the subgroup (SEC-IgG low, SEC-IgG high from IgG cells) mean of z-score of each gene expression from each donor.
