# Supplementary material for: SEC-seq: association of molecular signatures with antibody secretion in thousands of single human plasma cells
Source: Nat Commun. 2023 Jun 15;14:3567. doi: 10.1038/s41467-023-39367-8 (PMC10272111; doi:10.1038/s41467-023-39367-8)
Supplement: Supplementary file 3 — Reporting Summary [file 41467_2023_39367_MOESM3_ESM.pdf]

nature portfolio

Corresponding author(s): Richard James, Dino Di Carlo

Last updated by author(s): _2023/05/25

Reporting Summary

Nature Portfolio wishes to improve the reproducibility of the work that we publish. This form provides structure for consistency and transparency in reporting. For further information on Nature Portfolio policies, see our Editorial Policies and the Editorial Policy Checklist.

Statistics

For all statistical analyses, confirm that the following items are present in the figure legend, table legend, main text, or Methods section.

n/a	Confirmed	
☐	☒	The exact sample size (*n*) for each experimental group/condition, given as a discrete number and unit of measurement
☐	☒	A statement on whether measurements were taken from distinct samples or whether the same sample was measured repeatedly
☐	☒	The statistical test(s) used AND whether they are one- or two-sided<br>*Only common tests should be described solely by name; describe more complex techniques in the Methods section.*
☐	☒	A description of all covariates tested
☐	☒	A description of any assumptions or corrections, such as tests of normality and adjustment for multiple comparisons
☐	☒	A full description of the statistical parameters including central tendency (e.g. means) or other basic estimates (e.g. regression coefficient) AND variation (e.g. standard deviation) or associated estimates of uncertainty (e.g. confidence intervals)
☐	☒	For null hypothesis testing, the test statistic (e.g. *F*, *t*, *r*) with confidence intervals, effect sizes, degrees of freedom and *P* value noted<br>*Give P values as exact values whenever suitable.*
☒	☐	For Bayesian analysis, information on the choice of priors and Markov chain Monte Carlo settings
☒	☐	For hierarchical and complex designs, identification of the appropriate level for tests and full reporting of outcomes
☐	☒	Estimates of effect sizes (e.g. Cohen's *d*, Pearson's *r*), indicating how they were calculated

Our web collection on statistics for biologists contains articles on many of the points above.

Software and code

Policy information about availability of computer code

Data collection	dependency of python code analysis is in readme file. python version=3.8.12, read https://github.com/Rene2718/SEC-seq_plasma-cell_nanovial
Data analysis	Most clustering and gene differential analysis were using scanpy (l.8.2). Correlation analysis is using scipy(l.8.0). Heatmap plot is generated by seaborn(0 .11.2). Dataframe based analysis and table output is generated by pandas(l.4.2). Arrays and basic statistic (mean, sum) is calculated by numpy/pandas(l.21.5). Histogram and kernal-density plot are generated by matplotlib(3.5.l). Flow data were generated by flowjo (v10.8.1).

For manuscripts utilizing custom algorithms or software that are central to the research but not yet described in published literature, software must be made available to editors and reviewers. We strongly encourage code deposition in a community repository (e.g. GitHub). See the Nature Portfolio guidelines for submitting code & software for further information.

Data

Policy information about availability of data

All manuscripts must include a data availability statement. This statement should provide the following information, where applicable:

- Accession codes, unique identifiers, or web links for publicly available datasets
- A description of any restrictions on data availability
- For clinical datasets or third party data, please ensure that the statement adheres to our policy

Single cell RNA sequencing data files available on GEO (GSE229042). The samples were acquired from a commercial vendor under informed consent. The consent enabled distribution of RNA sequencing data. The IRB at Seattle Children's Research Institute deemed the experiments not human subjects. Consequently, there should be no hold ups regarding data availability. Raw data for figures are in "Source data" file.

Human research participants

Policy information about studies involving human research participants and Sex and Gender in Research.

Reporting on sex and gender	N/A- the research did not include human subjects as defined by NIH rules. Samples used were from a commercial source, de-identified and banked. However, the biological sex of the participants was described in manuscript. Neither sex, nor gender were considered in the study design or analysis.
Population characteristics	N/A- the research did not include human subjects as defined by NIH rules. Samples used were from a commercial source, de-identified and banked.
Recruitment	N/A- the research did not include human subjects as defined by NIH rules. Samples used were from a commercial source, de-identified and banked.
Ethics oversight	N/A- the research did not include human subjects as defined by NIH rules. Samples used were from a commercial source, de-identified and banked.

Note that full information on the approval of the study protocol must also be provided in the manuscript.

Field-specific reporting

Please select the one below that is the best fit for your research. If you are not sure, read the appropriate sections before making your selection.

☒ Life sciences ☐ Behavioural & social sciences ☐ Ecological, evolutionary & environmental sciences

For a reference copy of the document with all sections, see nature.com/documents/nr-reporting-summary-flat.pdf

Life sciences study design

All studies must disclose on these points even when the disclosure is negative.

Sample size	Sample size was calculated using power analyses prior to study initiation.
Data exclusions	No data was excluded in the manuscript.
Replication	Each experiment using human cells was replicated using at least three donors. flow cytometry data is having 7-8 replicated from 3-4 donors. and Image-flow /SEC-seq data is 3 replicates from 3 donors.
Randomization	The experiments in this paper involved monitoring B cells in nanovials. In each case bulk populations of cells were split based on phenotype (protein secretion) into control and/or experimental groups and tested separately. There was no a priori information available to make the groups non-random.
Blinding	For the Amnis imaging data, quantification and handling was done by different people than the lead scientist. The people performing quantification were blinded to the conditions (different donor). For flow cytometry, samples were coded during analysis and decoded after analysis. Main experiment SEC-seq is not blinded, due to single experiment is single condition (donor).

Reporting for specific materials, systems and methods

We require information from authors about some types of materials, experimental systems and methods used in many studies. Here, indicate whether each material, system or method listed is relevant to your study. If you are not sure if a list item applies to your research, read the appropriate section before selecting a response.

Materials & experimental systems

n/a	Involved in the study	
☐	☒	Antibodies
☐	☒	Eukaryotic cell lines
☒	☐	Palaeontology and archaeology
☒	☐	Animals and other organisms
☒	☐	Clinical data
☒	☐	Dual use research of concern

Methods

n/a	Involved in the study	
☒	☐	ChIP-seq
☐	☒	Flow cytometry
☒	☐	MRI-based neuroimaging

Antibodies

Antibodies used

Antibodies are detailed in Table S2

Validation

All antibodies used in this study for flow cytometry and/or CITEseq were purchased from Biolegend, abeam, or BD. We independently validate dilutions prior to use using culture conditions known to elicit staining in fixed percentages of cells in a heterogeneous mix. For antibodies against CD38, CD138, IgM, IgG, and CD19, we validated specificity using sgRNAs targeting the gene encoding the relevant proteins. CD45 antibodies were validated by demonstration of non-exclusive staining in mixed populations of human and mouse hematopoietic cells. For CITEseq studies, antibodies (IgG) were tested using fluorescent conjugates to determine dilutions that led to staining slightly above background. All antibodies used in this study are commercially available and validated by manufacture (we provided the main antibodies used with the link in below). https://www.biolegend.com/en-us/products/pe-cyanine7-anti-human-cd19-antibody-1911,https://www.fishersci.com/shop/products/cd38-mouse-anti-human-percp-cy5-5-clone-hit2-bd/BDB551400,https://www.biolegend.com/en-us/products/alexa-fluor-700-anti-human-cd138-syndecan-1-antibody-8957?GroupID=BLG15664,https://www.biolegend.com/en-us/products/pacific-blue-anti-human-igm-antibody-6637, https://www.bdbiosciences.com/en-us/products/reagents/flow-cytometry-reagents/research-reagents/single-color-antibodies-ruo/pe-mouse-anti-human-igg.555787

Eukaryotic cell lines

Policy information about cell lines and Sex and Gender in Research

Cell line source(s)

Primary human peripheral blood mononuclear cells were acquired from several donors using a commercial source. Raji Cells, CCL-86 (ATCC) https://www.atcc.org/products/ccl-86 .HyHel-5 hybridoma lines were provided by Richard Willson at University of Houston. Recieved from Cell culture company.

Authentication

Cells were analyzed by flow cytometry to confirm the presence of commonly present cell populations within PBMCs (B/T lymphocytes).

Mycoplasma contamination

Human PBMCs were not evaluated for mycoplasma. Contamination is not expected since these cells were frozen within 4 hours following their isolation from blood. Cell lines are periodically screened for Mycoplasma using the MycoAlert™ Mycoplasma Detection Kit.

Commonly misidentified lines
(See ICLAC register)

We did not use commonly misidentified cell lines.

Flow Cytometry

Plots

Confirm that:

☒ The axis labels state the marker and fluorochrome used (e.g. CD4-FITC).

☒ The axis scales are clearly visible. Include numbers along axes only for bottom left plot of group (a 'group' is an analysis of identical markers).

☒ All plots are contour plots with outliers or pseudocolor plots.

☒ A numerical value for number of cells or percentage (with statistics) is provided.

Methodology

Sample preparation

Single cells from culture or single cells encapsulated in nanovials were washed and resuspended in PBS. The cells were spun down and incubated with Fe-blocker and antibody cocktail for 20 minutes at 4C. For intracellular stains, we washed cells two times in PBS, fixed and permeabilized using the BD fixation/permeablization protocol and then stained as described above.

Instrument

BD LSR II flow cytometer, ARIA; Amnis ImageStream, and NanoSellect Wolf sorter.

Software

Flowjo

Cell population abundance

For the sorting studies, we assessed purity/abundance by analysis of the scRNA sequencing data.

Gating strategy

Prelimilary gating : FSC-A/SSC-A (cells)> FSC-A/FSC-H (singlet)> live-dead/SSC-A (live cell) Gating strategy: by contour plot with distinct population.

☒ Tick this box to confirm that a figure exemplifying the gating strategy is provided in the Supplementary Information.
